# Efficacy of Neoadjuvant Radiotherapy After Chemotherapy and the Optimal Interval from Radiotherapy to Surgery for Borderline Resectable and Resectable Pancreatic Cancer

**DOI:** 10.1245/s10434-024-16743-2

**Published:** 2025-01-14

**Authors:** Won-Gun Yun, Yoon Soo Chae, Youngmin Han, Hye-Sol Jung, Young Jae Cho, Hyun-Cheol Kang, Wooil Kwon, Joon Seong Park, Eui Kyu Chie, Jin-Young Jang

**Affiliations:** 1 Department of Surgery, Seoul National University College of Medicine, 101 Daehak-ro, Jongno-gu, Seoul, 03080 Republic of Korea; 2 Cancer Research Institute, Seoul National University College of Medicine, Seoul, Republic of Korea; 3 Department of Radiation Oncology, Seoul National University College of Medicine, 101 Daehak-ro, Jongno-gu, Seoul, 03080 Republic of Korea; 4 Institute of Radiation Medicine, Medical Research Center, Seoul National University, Seoul, Republic of Korea

**Keywords:** Pancreatic cancer, Neoadjuvant radiotherapy, Neoadjuvant chemotherapy, Pancreatectomy, Stereotactic body radiation therapy

## Abstract

**Background:**

Benefits of neoadjuvant treatment for pancreatic cancer with major vessel invasion has been demonstrated through randomized controlled trials; however, the optimal neoadjuvant treatment strategy remains controversial, especially for radiotherapy. Therefore, we aimed to evaluate the efficacy and safety of neoadjuvant radiotherapy followed by chemotherapy and the optimal time interval to undergo surgery after radiotherapy in (borderline) resectable pancreatic cancer.

**Methods:**

Between 2013 and 2022, patients with (borderline) resectable pancreatic cancer with vessel contact who received 5-fluorouracil with leucovorin, oxaliplatin, and irinotecan or gemcitabine and nanoparticle albumin-bound paclitaxel as initial treatment following surgery were included. Patients who received radiotherapy after chemotherapy and those who did not were matched using 1:1 nearest-neighbor propensity scores. Propensity scores were measured using the tumor size at initial image, duration of neoadjuvant chemotherapy, and responsiveness to neoadjuvant chemotherapy.

**Results:**

Of 212 patients, 166 patients were retrieved for the matched cohort. Patients who received radiotherapy had significantly better postoperative survival, local control, and R0 resection rates than those who did not. Furthermore, patients who underwent surgery within 4 weeks after completing radiotherapy had lower intraoperative blood loss and a clinically relevant postoperative pancreatic fistula rate than those who underwent surgery after more than 4 weeks.

**Conclusions:**

In patients with (borderline) resectable pancreatic cancer with vessel contact who were scheduled for curative-intent surgery after neoadjuvant chemotherapy, additional radiotherapy was associated with better postoperative survival and local control. Furthermore, our findings suggested that scheduling surgery within 4 weeks following radiation therapy might enhance the perioperative outcomes.

**Supplementary Information:**

The online version contains supplementary material available at 10.1245/s10434-024-16743-2.

Clinical trials have shown the benefits of neoadjuvant treatment for pancreatic cancer; nonetheless, the best treatment plan has been hotly debated. This study found that neoadjuvant radiotherapy added after chemotherapy was associated with improved prognosis in (borderline) resectable pancreatic cancer

Pancreatic ductal adenocarcinoma (PDAC) has an increasing annual incidence of 1% and an overall 5-year relative survival rate of 12%. It is expected PDAC will become the second biggest cause of cancer-related mortality by 2040, only surpassed by lung cancer.^1,2^ Although these statistics can be dismal at the first glance, developments in treatment strategies can be promising. First, advances in systemic treatment have been reported over the past few decades. Several randomized controlled trials have demonstrated the safety and efficacy of 5-fluorouracil with leucovorin, oxaliplatin, and irinotecan with or without dose modifications ([m]FOLFIRINOX) or gemcitabine with nanoparticle albumin-bound paclitaxel (GnP) in both metastatic and resected PDAC.^3–7^ Second, neoadjuvant treatment is increasingly being used for borderline resectable and resectable PDAC, which are considered as potentially curable. Neoadjuvant treatment has several theoretical advantages, such as treating micrometastasis early, facilitating a margin-negative operation, and selecting patients with favorable tumor biology to undergo pancreatectomy.^8^

Although the efficacy of neoadjuvant treatment in potentially curable PDAC has been demonstrated through several studies, an optimal neoadjuvant treatment regimen has not yet been defined.^9–13^ This is because there is limited evidence to recommend a specific neoadjuvant chemotherapy regimen. However, the National Comprehensive Cancer Network (NCCN) guidelines suggest (m)FOLFIRINOX or GnP as the preferred regimens in neoadjuvant treatment settings.^14^ Moreover, the role of neoadjuvant radiotherapy in PDAC is controversial. Although Jang et al. and the PREOPANC trial showed that neoadjuvant concurrent chemoradiation therapy (CCRT) with gemcitabine improves survival outcomes compared with upfront surgery in borderline resectable PDAC, only a few studies have compared neoadjuvant chemotherapy with or without radiotherapy.^9–11,15,16^

Other than surgery, radiotherapy is one of the techniques that has been demonstrated to improve local control by increasing the possibility of microscopically negative resection margins.^17^ Nonetheless, in previous reports, the radiation source and dose are varied and the results on its efficacy are inconsistent.^9–11,15,16^ Meanwhile, stereotactic body radiation therapy (SBRT) is an attractive approach for multimodality treatment in PDAC.^18–20^ The theoretical benefits of SBRT include the ability to deliver a high biologically effective dose and less interference with systemic treatment. However, the optimal SBRT scheme including the dose and fraction is not yet determined because of the radiosensitive organs surrounding the pancreas (i.e., duodenum, stomach, and colon). In addition, although radiotherapy-induced late tissue fibrosis can even increase the technical difficulty of surgery, there have been few studies regarding the optimal timing of surgery after completing neoadjuvant radiotherapy.^21,22^ Therefore, we aimed to evaluate the efficacy and safety of neoadjuvant radiotherapy followed by (m)FOLFIRINOX or GnP using propensity score matching and the optimal time interval to undergo surgery after radiotherapy in patients with borderline resectable or resectable PDAC.

## Methods

### Patient Cohort

This retrospective cohort study was approved by the Institutional Review Boards of Seoul National University Hospital (H-2401-116-1505) and was performed in accordance with the 1975 (and later versions) Declaration of Helsinki guidelines. This work was registered at Research Registry (researchregistry10160), and has been reported in line with the strengthening the reporting of cohort, cross-sectional, and case-control studies in surgery criteria.^23^

**Fig. 1:**
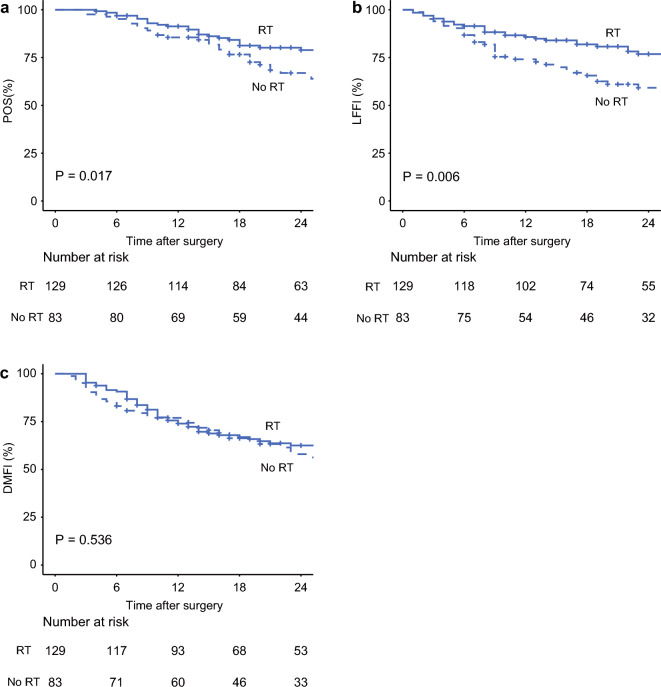
**a** POS, **b** LFFI, and **c** DMFI for patients who did or did not receive neoadjuvant radiotherapy in the unmatched cohort. *RT* radiotherapy, *POS* postoperative survival, *LFFI* locoregional failure-free interval, *DMFI* distant metastasis-free interval

Patients diagnosed with borderline resectable or resectable PDAC between 2013 and 2022, and who underwent curative-intent pancreatectomy after neoadjuvant (m)FOLFIRINOX or GnP, were included in the study. Radiologic resectability was determined based on the 2024 NCCN criteria defining resectability status at diagnosis.^14^ Patients who received total pancreatectomy (*n* = 36), had insufficient clinical information (*n* = 15), were identified as carbohydrate antigen (CA) 19-9 non-secretors (*n* = 13, defined as CA19-9 <2 on all preoperative examinations), and died within 3 months after surgery (*n* = 1) were excluded.^24^ Finally, 212 qualifying Asian patients were included and grouped according to whether they received neoadjuvant radiotherapy (electronic supplementary material [ESM] Fig. 1). The decision to proceed with and the type of neoadjuvant radiotherapy were made based on consolidated decisions by a multidisciplinary team. Radiotherapy options included CCRT (typically 56.0 Gy in 28 fractions) and SBRT (typically 50.0 Gy in 5 fractions).

### Data Collection

Patient demographics (including age, sex, body mass index, and physical status classification (based on the American Society of Anesthesiologists), tumor characteristics, and treatment specifics were collected. Tumor characteristics included location, size, and radiologic resectability at the time of diagnosis. Regarding treatment specifics, we evaluated the duration and responsiveness of neoadjuvant chemotherapy, which affected the prognosis. Duration of neoadjuvant chemotherapy was classified as short (≤3 months), intermediate (3–6 months), and long (>6 months). Responsiveness to neoadjuvant chemotherapy was classified as biochemical responders, radiologic only responders, and non-responders, in accordance with previously published research.^25^ Biochemical response to treatment was defined as CA19-9 after neoadjuvant chemotherapy within the normal range (≤37 U/mL), irrespective of the initial value, and radiologic response was defined as complete or partial response according to the Response Evaluation Criteria in Solid Tumors (RECIST) version 1.1.^26–28^

### Outcomes

The primary outcome was survival outcomes. Postoperative survival (POS) was defined from the date of pancreatectomy to the date of death, with censoring at the date of last follow-up for patients with no event. Locoregional failure-free interval (LFFI) and distant metastasis-free interval (DMFI) were measured from the date of pancreatectomy to the date of radiologic appearance of recurrent disease, with censoring at the date of last follow-up or death for patients with no event.

The secondary outcomes were microscopically negative (R0) resection rate, major pathologic response rate, and postoperative complications. Resection margin status was classified as R0 or R1 based on the 0 mm rule, and pathologic tumor regression was categorized based on the College of American Pathologists grading system (major: grade 0 or 1; minor: grade 2 or 3).^29–31^ Clavien–Dindo grade 3 or higher complications were collected, and the definition of clinically relevant postoperative pancreatic fistula (CR-POPF) followed that of the International Study Group of Pancreatic Surgery.^32,33^

### Subgroup Analysis

A subgroup analysis for patients who received neoadjuvant radiotherapy was conducted to compare the patients who received CCRT and those who received SBRT. A secondary subgroup analysis was performed to explore the optimal interval from the end of radiotherapy to surgery. Early surgery was defined as surgery within 4 weeks of radiotherapy ending, and late surgery was defined as surgery more than 4 weeks after radiotherapy had been completed. Although the best time to have surgery following radiotherapy has not yet been understood in pancreatic cancer, recent studies advise having surgery within 4–8 weeks after finishing radiotherapy; the policy at our center is the same.^15,34^ Thus, we established a cut-off of 4 weeks to ascertain the best timing for surgery within 8 weeks following the end of radiotherapy. Adverse events during neoadjuvant radiotherapy were graded using the Common Terminology Criteria for Adverse Events (CTCAE), version 4.0.^35^ Grade 4 or 5 adverse events were considered as severe events.

### Statistical Analysis

Variables were presented based on the treatment (neoadjuvant chemotherapy with or without radiotherapy) using descriptive statistics. Chi-square and Fisher’s exact tests were used to compare categorical variables and the independent t-test and Wilcoxon rank-sum test were used for continuous variables. Propensity score matching was performed using the 1:1 nearest-neighbor method with known prognostic factors such as tumor size at diagnosis, duration of neoadjuvant chemotherapy, and responsiveness to neoadjuvant chemotherapy. Survival analysis was performed using Kaplan–Meier estimates and compared using the log-rank test. Statistical significance was set at a *p*-value of <0.05, and marginal significance was set at a *p*-value of <0.10. All statistical analyses were conducted using R software, version 4.2.3 (The R Foundation for Statistical Computing, Vienna, Austria).

## Results

### Patient, Tumor, and Treatment Characteristics

Comparisons of the clinical characteristics between patients who received radiotherapy and those who did not are presented in Table 1. Before propensity score matching, 129 (60.8%) patients received radiotherapy and 83 (39.2%) did not. There were no differences in demographics except body mass index, tumor characteristics at the time of diagnosis, and adjuvant therapy, between patients who received radiotherapy and those who did not. However, the proportion of patients who received neoadjuvant chemotherapy for more than 6 months was significantly higher in patients who received radiotherapy than those who did not (31.0% vs. 15.7%, *p* < 0.001). In addition, the proportion of biochemical responders was marginally significantly higher in patients who received radiotherapy than those who did not (62.0% vs. 49.4%, *p* = 0.083).

**Table 1:** Clinical characteristics in the unmatched and matched cohorts

	RT (total)	RT (PSM)	No RT	*p*-value (total)	*p*-value (PSM)
Number	129	83	83		
Age, years [median (IQR)]	64 (59, 70)	64 (59, 70)	64 (58, 72)	0.651	0.680
Sex					
Male/female	64:65	41:42	43:40	0.864	0.877
BMI, kg/m^2^ [median (IQR)]	22.9	23.0	24.6	0.003	0.011
	(21.2, 24.5)	(21.0, 24.5)	(22.0, 26.5)		
ASA				0.543	0.641
I/II	110 (85.3)	71 (85.5)	74 (89.2)		
III/IV	19 (14.7)	12 (14.5)	9 (10.8)		
Location				0.841	0.612
Head	90 (69.8)	60 (72.3)	56 (67.5)		
Body/tail	39 (30.2)	23 (27.7)	27 (32.5)		
Tumor size, mm [median (IQR)]^a^	28 (24, 32)	30 (25, 35)	30 (24, 37)	0.107	0.893
Resectability^a^				0.992	>0.99
RPC	34 (26.4)	22 (26.5)	21 (25.3)		
BRPC	95 (73.6)	61 (73.5)	62 (74.7)		
Duration of NAC				<0.001	0.115
Short	17 (13.2)	17 (20.5)	29 (34.9)		
	72 (55.8)	50 (60.2)	41 (49.4)		
Long	40 (31.0)	16 (19.3)	13 (15.7)		
Response to NAC				0.083	0.783
BR+	80 (62.0)	43 (51.8)	41 (49.4)		
BR−/RR+	19 (14.7)	13 (15.7)	11 (13.3)		
BR−/RR−	30 (23.3)	27 (32.5)	31 (37.3)		
Adjuvant therapy				0.549	0.563
Yes	117 (90.7)	75 (90.4)	78 (94.0)		
No	12 (9.3)	8 (9.6)	5 (6.0)		

Data are expressed as *n* (%) unless otherwise specified

*RT* radiotherapy, *PSM* propensity score matching, *BMI* body mass index, *ASA* American Society of Anesthesiologists physical status classification, *(B)RPC* (borderline) resectable pancreatic cancer, *NAC* neoadjuvant chemotherapy, *BR* biochemical response, *RR* radiologic response, *IQR* interquartile range

^a^Description based on image at the time of diagnosis

After propensity score matching, 166 patients (83 in each group) were included. All patients who did not receive radiotherapy were included. Patients who received radiotherapy accounted to 64.3% of the unmatched cohort. There were no differences in the median tumor size at the time of diagnosis between patients who received radiotherapy and those who did not (30 mm vs. 30 mm, *p* = 0.893). In addition, there were no differences in the distribution of patients according to the duration of neoadjuvant chemotherapy, and in the responsiveness to neoadjuvant chemotherapy, between patients who received radiotherapy and those who did not. When comparing the post-matching standardized mean differences for matching variables with the pre-matching values, the overall trend was downward (ESM Fig. 2).

### Survival Outcomes

The Kaplan–Meier curves for POS, LFFI, and DMFI in the unmatched cohort are shown in Fig. 1. Patients who received radiotherapy showed a significantly higher 2-year POS rate than those who did not (78.9% vs. 66.9%, *p* = 0.017) (Fig. 1a). In addition, patients who received radiotherapy showed a significantly higher 2-year LFFI rate than those who did not (76.8% vs. 59.2%, *p* = 0.006) (Fig. 1b). However, there was no difference in the 2-year DMFI rate between patients who received radiotherapy and those who did not (62.5% vs. 58.0%, *p* = 0.536) (Fig. 1c).

**Fig. 2:**
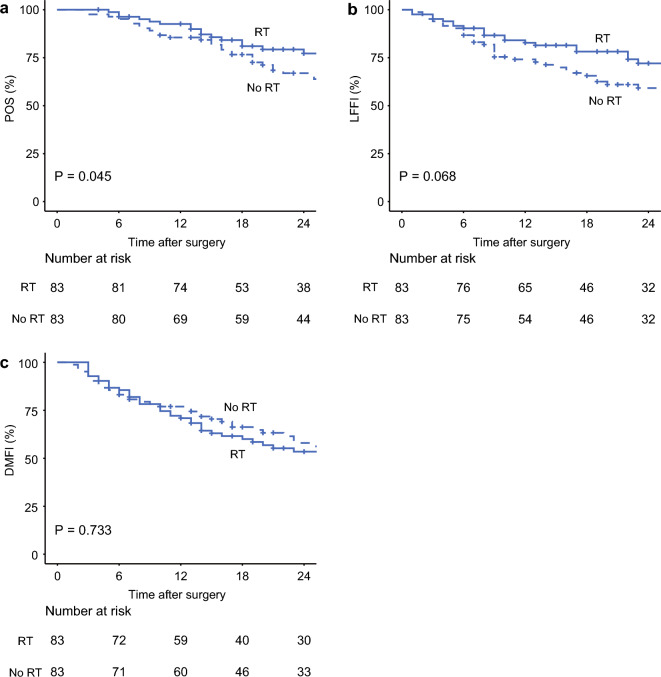
**a** POS, **b** LFFI, and **c** DMFI for patients who did or did not receive neoadjuvant radiotherapy in the matched cohort. *RT* radiotherapy, *POS* postoperative survival, *LFFI* locoregional failure-free interval, *DMFI* distant metastasis-free interval

The Kaplan–Meier curves for POS, LFFI, and DMFI in the matched cohort are shown in Fig. 2. The 2-year POS rate in the matched cohort was 77.2% with radiotherapy versus 66.9% without radiotherapy (*p* = 0.045) (Fig. 2a). In addition, the 2-year LFFI rate in the matched cohort was 72.1% with radiotherapy versus 59.2% without radiotherapy (*p* = 0.068) (Fig. 2b). Like the unmatched cohort, there was no difference in the 2-year DMFI rate between patients who received radiotherapy and those who did not (53.5% vs. 58.0%, *p* = 0.733) (Fig. 2c).

**Fig. 3:**
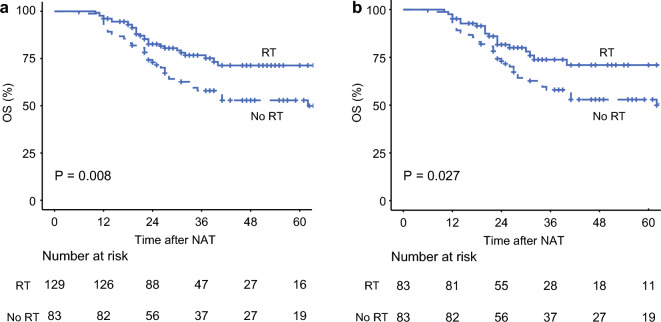
Overall survival from the start date of neoadjuvant treatment for patients with borderline resectable or resectable pancreatic cancer who did or did not receive neoadjuvant radiotherapy in the **a** unmatched and **b** matched cohorts. *RT* radiotherapy, *OS* overall survival, *NAT* neoadjuvant treatment

Overall survival from the start date of neoadjuvant treatment was also estimated in both the unmatched and matched cohorts. A 2-year (5-year) overall survival rate in the unmatched cohort was 82.6% (71.3%) with radiotherapy versus 73.0% (52.9%) without radiotherapy (*p* = 0.008) (Fig. 3a). In line with the unmatched cohort, the 2-year (5-year) overall survival rate in the matched cohort was 81.7% (71.0%) with radiotherapy versus 73.0% (52.9%) without radiotherapy (*p* = 0.027) (Fig. 3b).

### Clinical and Pathologic Outcomes in the Matched Cohort

Clinical and pathologic outcomes in the matched cohort are presented in Table 2. Patients who received radiotherapy showed a marginally significantly higher R0 resection rate than those who did not (92.8% vs. 81.9%, *p* = 0.062). However, there were no differences in node-negativity and major pathologic response rates between the two groups. Additionally, there were no differences in postoperative complication and CR-POPF rates between the two groups.

**Table 2:** Clinical and pathological outcomes based on the receipt of neoadjuvant radiotherapy in the matched cohort

	Total	RT (PSM)	No RT	*p*-value
Number	166	83	83	
Operation				0.335
PD	113 (68.1)	58 (69.9)	55 (66.3)	
DP	46 (27.7)	20 (24.1)	26 (31.3)	
STP	7 (4.2)	5 (6.0)	2 (2.4)	
Method				0.064
Open	158 (95.2)	76 (91.6)	82 (98.8)	
MIS	8 (4.8)	7 (8.4)	1 (1.2)	
Vessel resection				0.867
Yes	52 (31.3)	25 (30.1)	27 (32.5)	
No	114 (68.7)	58 (69.9)	56 (67.5)	
Operation time, min [median (IQR)]	260 (200, 320)	240 (195, 299)	275 (215, 355)	0.006
Blood loss, mL [median (IQR)]	500 (300, 800)	500 (325, 800)	500 (300, 800)	0.948
Margin status				0.062
R0	145 (87.3)	77 (92.8)	68 (81.9)	
R1	21 (12.7)	6 (7.2)	15 (18.1)	
T stage				0.175
ypT0	7 (4.2)	6 (7.2)	1 (1.2)	
ypT1-2	136 (81.9)	65 (78.3)	71 (85.5)	
ypT3-4	23 (13.9)	12 (14.5)	11 (13.3)	
N stage				0.640
ypN0	92 (55.4)	48 (57.8)	44 (53.0)	
ypN+	74 (44.6)	35 (42.2)	39 (47.0)	
Pathologic response				>0.99
Major	39 (23.5)	19 (22.9)	20 (24.1)	
Minor	127 (76.5)	64 (77.1)	63 (75.9)	
Lymphatic invasion, yes	43 (25.9)	22 (26.5)	21 (25.3)	>0.99
Venous invasion, yes	53 (31.9)	31 (37.3)	22 (26.5)	0.183
Perineural invasion, yes	118 (71.1)	59 (71.1)	59 (71.1)	>0.99
Complication^a^	28 (16.9)	17 (20.5)	11 (13.3)	0.300
CR-POPF	8 (4.8)	5 (6.0)	3 (3.6)	0.720

Data are expressed as *n* (%) unless otherwise specified

*RT* radiotherapy, *PSM* propensity score matching, *PD* pancreaticoduodenectomy, *DP* distal pancreatectomy, *STP* subtotal pancreatectomy, *MIS* minimally invasive surgery, *CR-POPF* clinically relevant postoperative pancreatic fistula

^a^Clavien–Dindo grade ≥3

### Details on Radiation Therapy

Details according to the type of radiotherapy are presented in ESM Table 1. Of the 129 patients who received neoadjuvant radiotherapy, 12 (9.3%) received CCRT and 117 (90.7%) received SBRT. A median radiation dose was 56.0 Gy with CCRT versus 50.0 Gy with SBRT (*p* < 0.001). The median fraction was 28 with CCRT versus 5 with SBRT (*p* < 0.001). The standard prescription for CCRT was 56.0 Gy to the primary tumor and 50.4 Gy to the elective nodal station, in 28 fractions using a simultaneous integrated boost. In contrast, for SBRT, the standard prescription was 50.0 Gy to the primary tumor, excluding the planning organ at risk volume, and 25.0–35.0 Gy to the planning organ at risk volume, using isotoxic simultaneous integrated protection. There were no differences in all adverse events between patients who received CCRT and those who received SBRT (16.7% vs. 7.7%, *p* = 0.272). None of the patients in either group exhibited clinically severe grade 4 or higher adverse events.

The Kaplan–Meier curve for POS, according to the type of radiotherapy, is shown in ESM Fig. 3. There were no differences in the 2-year POS rate between patients who received CCRT and those who received SBRT (66.7% vs. 80.8%, *p* = 0.700).

### Optimal Interval from Radiotherapy to Surgery

Clinical outcomes according to the interval from radiotherapy to surgery are presented in Table 3. The median (interquartile range) interval from radiotherapy to surgery was 1.6 (1.0–2.6) weeks. Of the 129 patients who received neoadjuvant radiotherapy, 111 (86.0%) underwent surgery within 4 weeks after radiotherapy, and 18 (14.0%) underwent surgery more than 4 weeks after radiotherapy. There were no significant differences in operation type and method between the two groups. However, the intraoperative blood loss was higher in the late surgery group than in the early surgery group (850 mL vs. 450 mL, *p* = 0.019). Additionally, the late surgery group showed a higher CR-POPF rate than the early surgery group (16.7% vs. 3.6%, *p* = 0.056). The late surgery group also showed higher vessel resection and postoperative complication rates compared with the early surgery group, but these differences did not reach statistical significance. In addition, among patients who underwent major vessel resection, there were no differences in the rate of tumor invasion to the major vessel between the early (35.3%) and late (37.5%) surgery groups (*p* > 0.99). However, in terms of oncologic feasibility, there were no differences in the R0 resection rate, major pathologic response rate, and locoregional failure rate between the two groups.

**Table 3:** Clinical outcomes according to the interval from the end date of radiotherapy to surgery

	Early (<4 weeks)	Late (≥4 weeks)	*p*-Value
Number	111	18	
Operation			0.354
PD	77 (69.4)	4 (22.2)	
DP	30 (27.0)	12 (66.7)	
STP	4 (3.6)	2 (11.1)	
Method			>0.99
Open	103 (92.8)	17 (94.4)	
MIS	8 (7.2)	1 (5.6)	
Vessel resection			0.283
Yes	34 (30.6)	8 (44.4)	
No	77 (69.4)	10 (55.6)	
Operation time, min [median (IQR)]	240 (188, 290)	240 (208, 306)	0.434
Blood loss, mL [median (IQR)]	450 (300, 750)	850 (425, 1183)	0.019
Margin status			0.373
R0	102 (91.9)	15 (83.3)	
R1	9 (8.1)	3 (16.7)	
Pathologic vessel invasion^a^			>0.99
Yes	12 (35.3)	3 (37.5)	
No	22 (64.7)	5 (62.5)	
Pathologic response			0.242
Major	26 (23.4)	7 (38.9)	
Minor	85 (76.6)	11 (61.1)	
Locoregional failure	24 (21.6)	6 (33.3)	0.365
Complication^b^	18 (16.2)	5 (27.8)	0.315
All POPF	17 (15.3)	8 (44.4)	0.008
CR-POPF	4 (3.6)	3 (16.7)	0.056

Data are expressed as *n* (%) unless otherwise specified

*PD* pancreaticoduodenectomy, *DP* distal pancreatectomy, *STP* subtotal pancreatectomy, *MIS* minimally invasive surgery, *POPF* postoperative pancreatic fistula, *CR-POPF* clinically relevant postoperative pancreatic fistula

^a^Patients who underwent major vessel resection during pancreatectomy

^b^Clavien–Dindo grade ≥3

## Discussion

Although neoadjuvant radiotherapy has theoretical advantages because of its effect on increased R0 resection rates and enhanced tumor response by administering well-oxygenated cells before surgery, the benefit of radiotherapy remains controversial in PDAC.^36^ Using a propensity score-matched analysis of 166 patients with borderline resectable or resectable PDAC with vessel contact who received (m)FOLFIRINOX or GnP as initial treatment, this study showed that the 2-year POS rate with radiotherapy was 77.2% (2- and 5-year overall survival rate from the start date of neoadjuvant treatment of 81.7% and 71.0%, respectively) compared with 66.9% (73.0% and 52.9%, respectively) without radiotherapy. This difference was maintained in local control (2-year LFFI: 72.1% vs. 59.2%, *p* = 0.068) and R0 resection rates (92.8% vs. 81.9%, *p* = 0.062). In addition, we also found that patients who received surgery within 4 weeks after the end of radiotherapy showed better clinical outcomes, including intraoperative blood loss and CR-POPF rate, than those who received surgery 4 weeks or later after radiotherapy.

The use of radiotherapy in combination with chemotherapy in the neoadjuvant setting remains an open question. Although several studies have compared neoadjuvant treatment and immediate surgery for borderline resectable and resectable PDAC, very few studies have compared neoadjuvant chemotherapy with or without radiotherapy.^9–13^ The Alliance A021501 trial showed that the 18-month OS rate was 66.4% with neoadjuvant mFOLFIRINOX alone versus 47.3% with neoadjuvant mFOLFIRINOX followed by hypofractionated image-guided radiotherapy (25 Gy in 5 fractions) or SBRT (33–40 Gy in 5 fractions).^15^ Data collection for the neoadjuvant mFOLFIRINOX followed by radiotherapy group was closed after 56 patients due to an interim analysis demonstrating futility regarding the R0 resection rate. In a multicenter retrospective study conducted by the Trans-Atlantic Pancreatic Surgery Consortium (TAPS), there were no differences in overall survival between patients who underwent neoadjuvant (m)FOLFIRINOX with or without radiotherapy in both the resected and non-resected cohorts.^16^ However, this retrospective study demonstrated the efficacy of neoadjuvant radiotherapy, and the results were different to that shown in previous research. The first distinction between this study and previous research is the radiation dose. In our institution, median radiation dose (biologically effective dose; α/β = 10) was 56.0 (67.2) Gy for CCRT and 50.0 (100.0) Gy for SBRT. However, in the previous study, typical radiation dose (biologically effective dose; α/β = 10) was 50.4 (59.5) Gy for CCRT and lower than 40.0 (72.0) Gy for SBRT. Several retrospective studies have demonstrated that a high radiation dose in CCRT was associated with improved survival outcomes in patients with locally advanced PDAC.^37,38^ Regarding SBRT, a phase I trial of radiation dose escalation showed favorable local control and survival outcomes in all-stage PDAC, and a recent phase Ib/II randomized controlled trial conducted by Taniguchi et al. demonstrated the tolerance of SBRT that used 50 or 55 Gy in 5 fractions for patients with localized PDAC.^39,40^ The second distinction is the duration of neoadjuvant chemotherapy and cohort composition. According to the study design, patients in the Alliance A021501 trial received neoadjuvant (m)FOLFIRINOX chemotherapy for 4 months, and for 2 months in the ESPAC-5 trial.^13,15^ In a retrospective study by the TAPS Consortium, <15% of patients received neoadjuvant (m)FOLFIRINOX chemotherapy for more than 4 months.^16^ In this study, 128/212 (60.4%) patients received neoadjuvant chemotherapy for more than 4 months, and only patients who underwent curative resection were included. These findings suggested that the efficacy of local treatment such as radiotherapy can be maximized when adequate systemic control is attained. Conversely, this could also imply that individuals who were supposed to undergo surgery after receiving adequate chemotherapy could be a subpopulation that may benefit from addition of radiotherapy. In addition, radiotherapy effects have been improved by improving radiation dose localization and reducing peripheral normal tissue damage. The feasibility and tolerability of advanced radiotherapy, such as stereotactic magnetic resonance-guided adaptive radiation therapy or carbon-ion radiation therapy, has been demonstrated in patients with PDAC.^41,42^ Future studies on the synergistic effects of modern chemotherapy regimens and cutting-edge radiotherapy in the neoadjuvant setting are required for clinical application.

Concerns have been expressed by several surgeons that radiotherapy before surgery could lead to surgical difficulties and postoperative complications. However, the study by Jang et al. (2018), as well as the PREOPANC trial, showed that there were no significant differences in surgical complications between patients who received neoadjuvant CCRT and those who underwent immediate surgery.^9–11^ Additionally, in the ESPAC-5 trial, there were no significant differences in surgical complications between patients who received neoadjuvant treatment including chemotherapy alone or CCRT and those who underwent immediate surgery.^13^ In this study, similar to previous studies, there were no differences in intraoperative blood loss and postoperative complications between patients who underwent neoadjuvant chemotherapy with or without radiotherapy. This study goes a step further by evaluating the best time to schedule surgery after completing radiotherapy. In comparison with patients who underwent surgery within 4 weeks after finishing radiotherapy, those who had surgery after 4 weeks or later experienced higher blood loss during surgery and more frequent CR-POPF. These findings could be related to the timing of pancreas tissue damage induced by radiation (such as fibrosis).^43,44^ However, there was no significant association between the interval from radiotherapy to surgery and oncologic outcomes, including R0 resection, major pathologic response, and local control rates, unlike rectal cancer, where delayed surgery has been reported to be associated with a higher degree of tumor control.^45,46^ Therefore, if feasible, we typically schedule surgery between 2 and 4 weeks following radiotherapy because it is likely that tissue damage (inflammation and edema) might occur in the early stages after radiotherapy.^44^

Several limitations of this study need to be acknowledged. First, our results could be subjected to confounding by indication because the patients who received neoadjuvant radiotherapy received a longer duration of chemotherapy and showed more favorable response to chemotherapy. Propensity score-matched analysis was used to deal with these biases, but residual bias from unmeasured factors might still exist. Second, although a comparison between patients who received neoadjuvant CCRT and those who received SBRT was performed, the number of patients who underwent CCRT was relatively limited. Third, this study only included patients who underwent surgery after neoadjuvant treatment, excluding those who did not undergo surgery due to cancer progression during neoadjuvant treatment. This suggests the possibility of selection bias. Fourth, since the duration of neoadjuvant treatment varies among patients, analyzing POS may result in immortal time bias. Fifth, bias may arise from the fact that the methods used to assess independent variables were employed to compare the two groups (radiotherapy group vs. no radiotherapy group) in the matched cohort.

## Conclusion

This study demonstrated that neoadjuvant radiotherapy was associated with improved 2-year POS, local control, and R0 resection rates in patients with borderline resectable or resectable PDAC who were scheduled for pancreatectomy after (m)FOLFIRINOX or GnP. Furthermore, our findings suggest that scheduling surgery within 4 weeks after completing radiotherapy could enhance the operative outcomes, including intraoperative blood loss and CR-POPF.

## Supplementary Information

Below is the link to the electronic supplementary material.

Supplementary file1 (DOCX 564 kb)
