# Supplementary material for: Efficacy of Neoadjuvant Radiotherapy After Chemotherapy and the Optimal Interval from Radiotherapy to Surgery for Borderline Resectable and Resectable Pancreatic Cancer
Source: Ann Surg Oncol. 2025 Jan 14;32(4):2819–29. doi: 10.1245/s10434-024-16743-2 (PMC11882644; doi:10.1245/s10434-024-16743-2)

**Supplementary Materials - Index**

| **Supplementary Figures and Tables** |  |
| --- | --- |
| Supplementary Figure 1 | page. 2 |
| Supplementary Figure 2 | page. 3 |
| Supplementary Table 1 | page. 4 |
| Supplementary Figure 3 | page. 5 |

**Supplementary FIG. 1** Flow chart of this study. *(B)RPC* (borderline) resectable pancreatic cancer, *(m)FOLFIRINOX* (modified) 5-fluorouracil, leucovorin, irinotecan, and oxaliplatin, *GnP* gemcitabine with nanoparticle albumin-bound paclitaxel, *CA 19-9* carbohydrate antigen 19-9


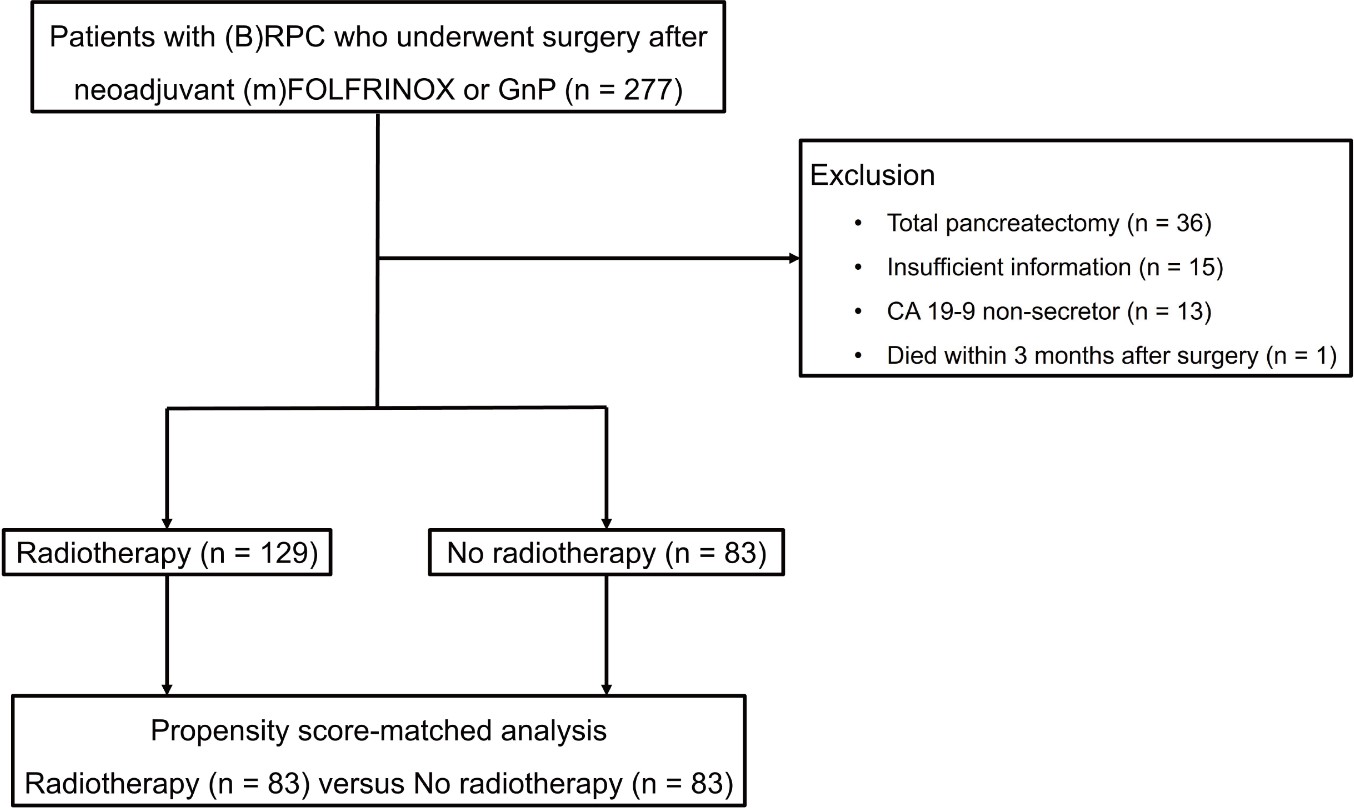


**Supplementary FIG. 2** Statistical differences in variables between patients who underwent neoadjuvant radiotherapy and those who did not undergo before and after propensity-score matching. *BR* biochemical response, *RR* radiologic response


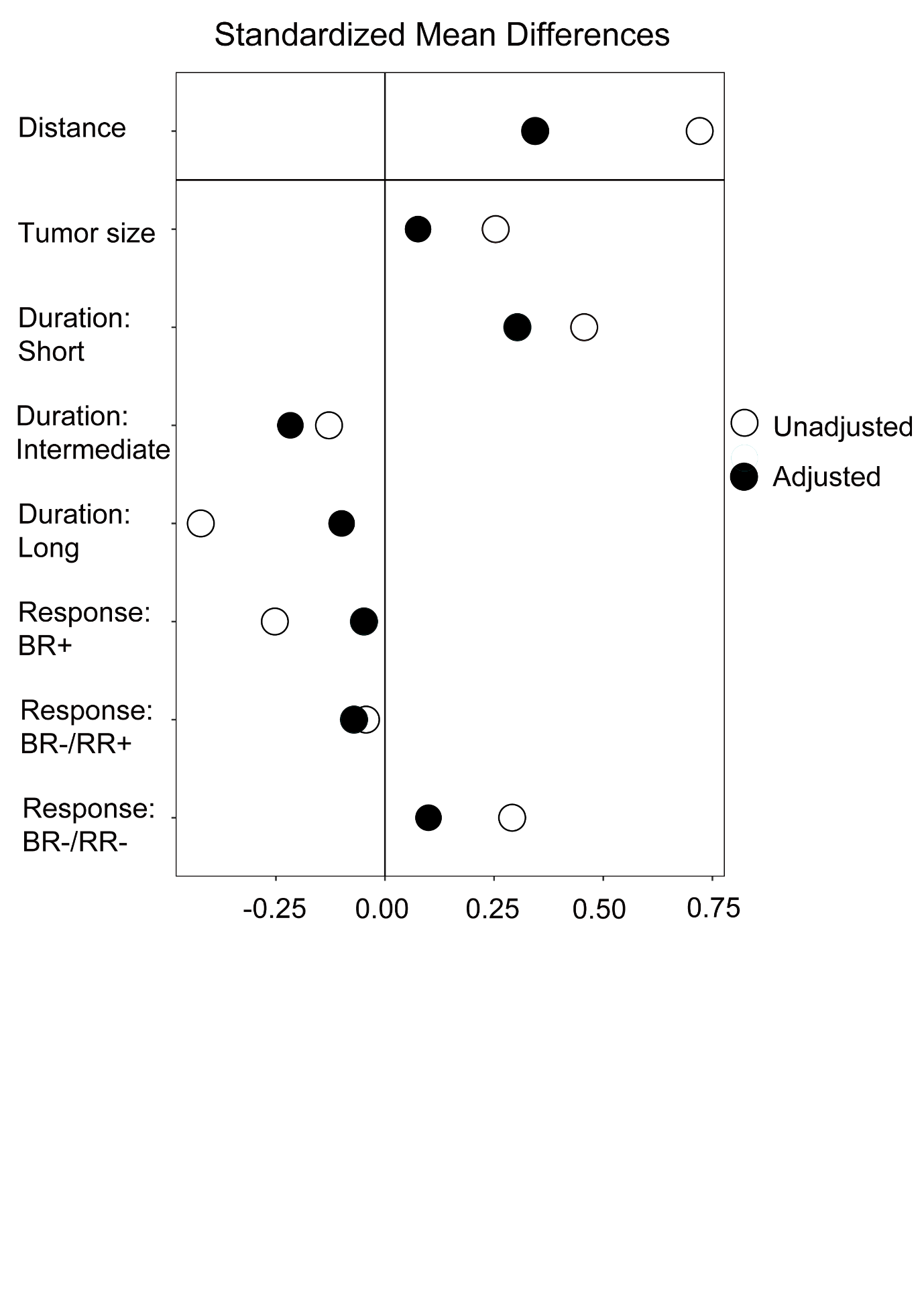


**Supplementary TABLE 1** Details about radiotherapy according to the type of radiotherapy

|  | **CCRT** | **SBRT** | **P** |
| --- | --- | --- | --- |
| Number | 12 | 117 |  |
| Radiation dose (Gy)^†^ | 56.0 (55.5, 56.0) | 50.0 (48.0, 50.0) | < 0.001 |
| Number of Fractions | 28 (28, 28) | 5 (5, 5) | < 0.001 |
| Radiosensitizer |  |  | Not applicable |
| 5-fluorouracil | 6 (50.0) | 0 (0.0) |  |
| Gemcitabine | 4 (33.3) | 0 (0.0) |  |
| Capecitabine | 2 (16.7) | 0 (0.0) |  |
| N | 0 (0.0) | 117 (100.0) |  |
| AEs (All)^¶^ | 2 (16.7) | 9 (7.7) | 0.272 |
| AEs (Grade ≥ 4)^¶^ | 0 (0.0) | 0 (0.0) | Not applicable |

*CCRT* concurrent chemoradiation therapy, *SBRT* stereotactic body radiation therapy, *AE* adverse event

Values in parentheses are percentages unless indicated otherwise.

^†^Median values with interquartile range.

^¶^Based on Common Terminology Criteria for Adverse Events version 4.0.

**Supplementary FIG. 3** Postoperative survival for patients with borderline resectable or resectable pancreatic cancer who received neoadjuvant CCRT versus SBRT after neoadjuvant chemotherapy. *CCRT* concurrent chemoradiation therapy, *SBRT* stereotactic body radiation therapy, *POS* postoperative survival


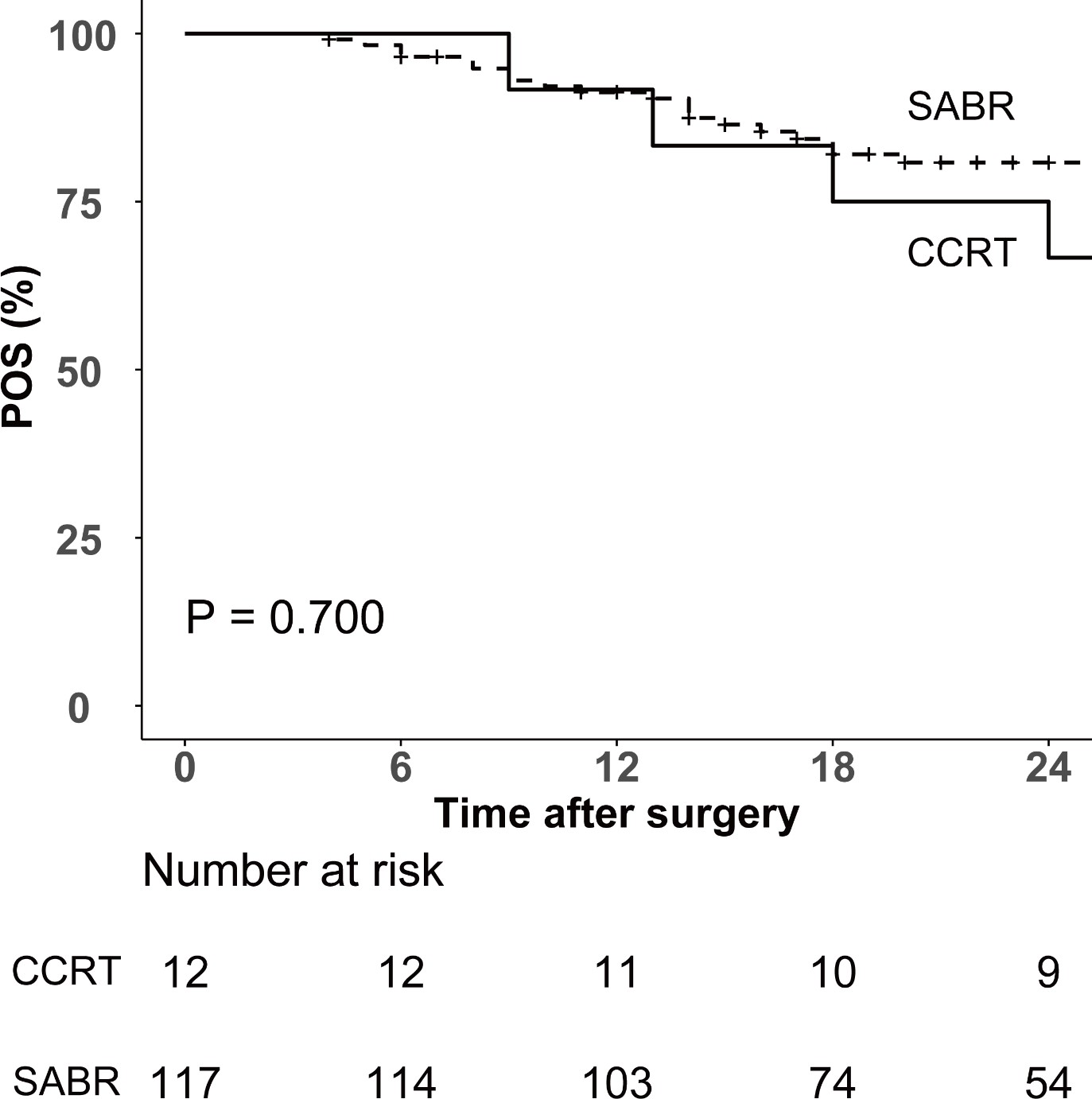

Supplement: Supplementary file 1 — Supplementary file1 (DOCX 564 kb) [file 10434_2024_16743_MOESM1_ESM.docx]
